# Draft Genome Sequence of the Hydrocarbon-Degrading Bacterium *Acinetobacter pittii* Strain ABC Isolated from Noonmati Refinery, Assam, India

**DOI:** 10.1128/genomeA.01264-17

**Published:** 2017-11-02

**Authors:** Arvind Kumar Singh, Bobby Chettri, Arpita Ghosh, Surendra K. Chikara, Timir Tripathi

**Affiliations:** a Department of Biochemistry, North Eastern Hill University, Shillong, India; b Eurofins Genomics, Doddanakundi Industrial Area 2, Seetharampalya, Hoodi, Bengaluru, Karnataka, India

## Abstract

We report here the 3.84-Mb draft genome sequence of hydrocarbon-degrading *Acinetobacter pittii* strain ABC isolated from oil-contaminated soil in Guwahati, India. The genome sequence contains 3,602 coding sequences and a G+C content of 38.83%. This is the first report of the genome sequence of an *Acinetobacter pittii* from an oil-contaminated environment.

## GENOME ANNOUNCEMENT

Bacteria of the genus *Acinetobacter* are strictly aerobic, nonfermentative, Gram-negative bacilli that are ubiquitously distributed in nature, including in hydrocarbon-contaminated environments (1). Previous studies have shown the degradation of different hydrocarbons by *Acinetobacter* (1–4). A single report on the complete genome sequence of *Acinetobacter pittii* is available in the database (Refseq no. NC_016603.1). There is no information on the complete genome sequence of this organism from crude oil-polluted sediments. Here we report the complete genome sequence of *Acinetobacter pittii* strain ABC, an isolate from crude oil-contaminated sediment collected from Noonmati refinery, Assam, India. The isolate exhibited a wide range of substrate specificity for both aliphatic and aromatic hydrocarbons as sole sources of carbon and energy.

The genome sequence of *Acinetobacter pittii* strain ABC was sequenced by use of the Illumina NextSeq 500 (paired-end library 2 × 150 bp) to generate 623 Mb of data. *De novo* assembly was carried out using Velvet v1.2.10 (5) to generate 32 scaffolds contributing to 3.84 Mb of the draft genome. The *N*_50_ value was 365,839 bp, and an average scaffold length of 120,103 bp depicted the assembly quality. The genome was estimated to have an overall G+C content of 38.83%. Prodigal v2.50 was used to identify 3,602 genes by using the unsupervised machine learning algorithm, with an average length of 313 amino acids (6). Blast2Go was used to annotate the predicted genes.

*Acinetobacter pittii* strain ABC demonstrates the presence of several hydrocarbon-degrading genes and genes related to this process, such as dioxygenases, rubredoxin, esterase, alkane-1-monooxygenase, alkane sulfonate monooxygenase, 4-carboxymuconolactone decarboxylase, 3-carboxy-*cis*, *cis*-muconate cycloisomerase, 3-oxoadipate-enol-lactonase, protocatechuate 3, and salicylaldehyde dehydrogenase. Information about the genome sequence of *Acinetobacter pittii* strain ABC will be helpful in understanding the diversity and mechanisms of hydrocarbon degradation in the crude oil-polluted sediments and bioremediation technologies (7–9).

### Accession number(s).

This whole-genome shotgun project of *Acinetobacter pittii* strain ABC has been deposited at DDBJ/ENA/GenBank under the accession number MSPO00000000.
